# The Effect of Single Nucleotide Variations in the Transmembrane Domain of OATP1B1 on *in vitro* Functionality

**DOI:** 10.1007/s11095-021-03107-8

**Published:** 2021-10-13

**Authors:** Wilma Kiander, Kati-Sisko Vellonen, Melina M. Malinen, Mikko Gynther, Marja Hagström, Madhushree Bhattacharya, Seppo Auriola, Jan B. Koenderink, Heidi Kidron

**Affiliations:** 1 Division of Pharmaceutical Biosciences, Faculty of Pharmacy, University of Helsinki, Viikinkaari 5E, 00014 Helsinki, Finland; 2 School of Pharmacy, University of Eastern Finland, Kuopio, Finland; 3 Pharmacy and Molecular Biotechnology, Ruprecht-Karls-University, Heidelberg, Germany; 4 Department of Pharmacology and Toxicology, Radboud Institute for Molecular Life Sciences, Radboud University Medical Center, Nijmegen, the Netherlands; 5 Division of Pharmaceutical Biosciences, Faculty of Pharmacy, University of Helsinki, Viikinkaari 5E, 00014 Helsinki, Finland

## Abstract

**Purpose:**

Organic Anion Transporting Polypeptide 1B1 (OATP1B1) mediates hepatic influx and clearance of many drugs, including statins. The *SLCO1B1* gene is highly polymorphic and its function-impairing variants can predispose patients to adverse effects. The effects of rare genetic variants of *SLCO1B1* are mainly unexplored. We examined the impact of eight naturally occurring rare variants and the well-known *SLCO1B1* c.521C > T (V174A) variant on *in vitro* transport activity, cellular localization and abundance.

**Methods:**

Transport of rosuvastatin and 2,7-dichlorofluorescein (DCF) in OATP1B1 expressing HEK293 cells was measured to assess changes in activity of the variants. Immunofluorescence and confocal microscopy determined the cellular localization of OATP1B1 and LC–MS/MS based quantitative targeted absolute proteomics analysis quantified the amount of OATP1B1 in crude membrane fractions.

**Results:**

All studied variants, with the exception of P336R, reduced protein abundance to varying degree. V174A reduced protein abundance the most, over 90% compared to wild type. Transport function was lost in G76E, V174A, L193R and R580Q variants. R181C decreased activity significantly, while T345M and L543W retained most of wild type OATP1B1 activity. P336R showed increased activity and H575L decreased the transport of DCF significantly, but not of rosuvastatin. Decreased activity was interrelated with lower absolute protein abundance in the studied variants.

**Conclusions:**

Transmembrane helices 2, 4 and 11 appear to be crucial for proper membrane localization and function of OATP1B1. Four of the studied variants were identified as loss-of-function variants and as such could make the individual harboring these variants susceptible to altered pharmacokinetics and adverse effects of substrate drugs.

**Supplementary Information:**

The online version contains supplementary material available at 10.1007/s11095-021-03107-8.

## Introduction

Organic Anion Transporting Polypeptide 1B1 (OATP1B1; encoded by *SLCO1B1*), a sinusoidal hepatic uptake transporter, plays a critical role in the pharmacokinetics of many widely used drugs such as cholesterol-lowering statins (1). EMA and FDA have issued guidelines on its study on pharmacokinetics during drug development, recognizing the importance of OATP1B1 in drug safety (2, 3).

Single nucleotide variations (SNV) in the *SLCO1B1* gene can reduce plasma membrane expression or alter substrate affinity to the transporter, leading to altered pharmacokinetics of its substrate drugs. The well-characterized c.521 T > C SNV reduces clearance and elevates plasma concentrations of many statins (4). Results of this can, in a dose-dependent manner, be muscle pain, weakness, and, in the worst-case, rhabdomyolysis. Compared to non-carriers, homozygous carriers of the c.521 T > C variant are 30 times more susceptible to the development of simvastatin-induced myopathy (5).

The *SLCO1B1* c.521 T > C variant is rather common: 32% of people with European ancestry are heterozygotes (TC genotype) and 4% are homozygotes (CC genotype) (6). This has enabled clinical association studies with good statistical power. With rare genetic variants, it is difficult to gather a patient group of sufficient size; nonetheless, their impact on drug safety can be as consequential (7). To overcome this issue, methods in *in vitro—in vivo* extrapolation (IVIVE) such physiologically based pharmacokinetic modelling (PBPK) incorporating *in vitro* data can help estimate the changes in *in vivo* pharmacokinetics and identify genotypes that could warrant caution when prescribing OATP1B1 substrate drugs (8).

The three-dimensional molecular structure of OATP1B1 has yet to be established and knowledge on the important substrate-binding sites is limited. OATP1B1 is predicted to form twelve transmembrane helices, with cytoplasmic N- and C-terminals. Site-directed mutation studies have provided information on the effects of amino acid substitution on transporter function and identified transmembrane helices that are essential for transporter function (9). Based on computational predictions tools such as SIFT and PolyPhen, many of the naturally occurring genetics variants in the putative transmembrane region are deleterious (10, 11).

**Fig. 1:**
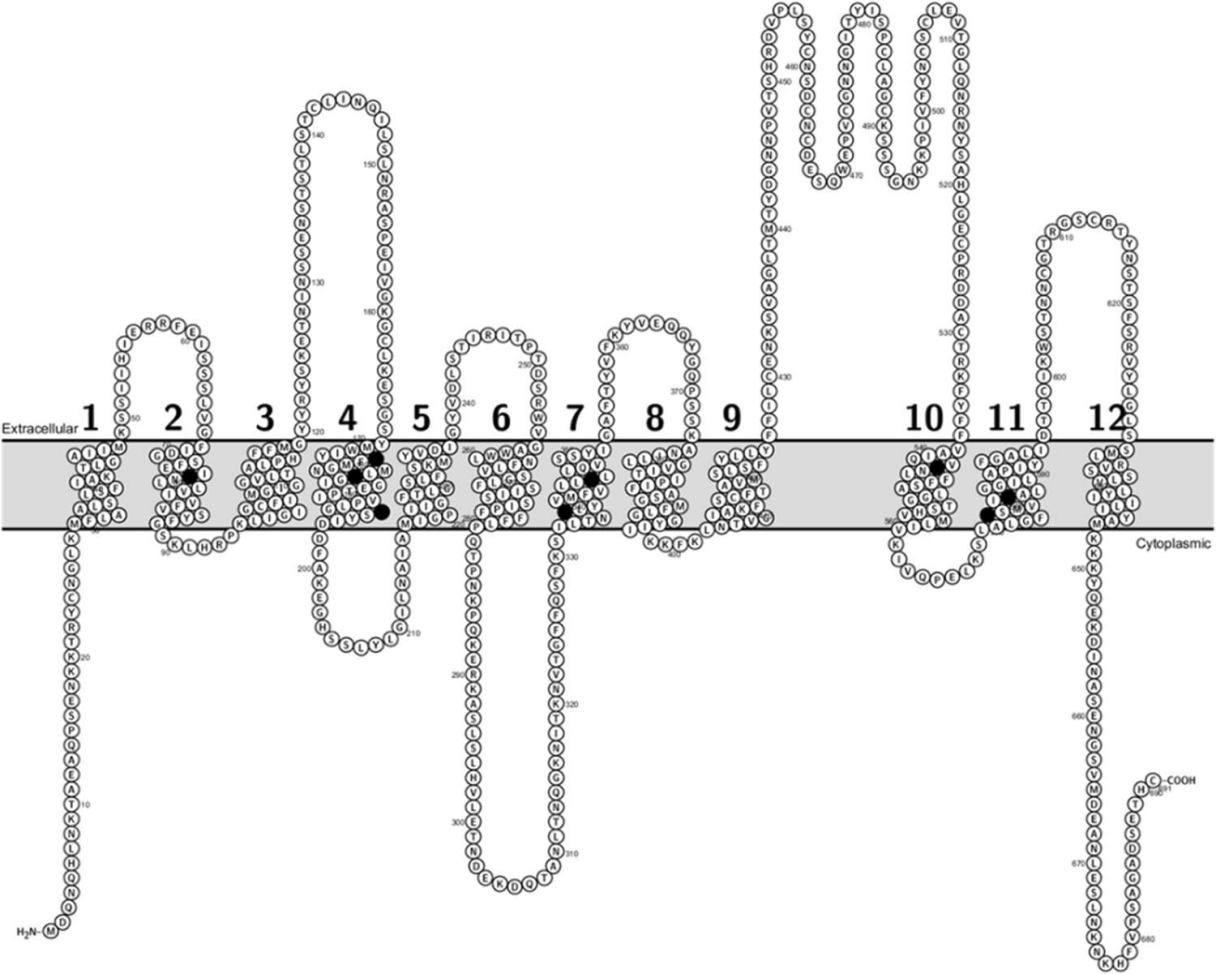
Location of the selected single nucleotide variants in the predicted transmembrane helices of OATP1B1. The figure is based on the Uniprot entry Q9Y6L6 and generated with Protter (12)

Here we characterized nine naturally occurring SNVs, where the amino acid substitution occurs in the putative transmembrane region of OATP1B1 (Fig. 1, Table I). The variants were chosen based on the changes in the characteristics of primary protein structure such as loss or gain of charge and location in critical transmembrane helices. We determined the cellular uptake of two OATP1B1 substrates, 2,7-dichlorofluorescein (DCF) and rosuvastatin, in OATP1B1 overexpressing HEK293 cells, and evaluated the cellular localization and quantified the protein expression of the variants. The *SLCO1B1* c.521 T > C SNV served as a clinical reference variant. Our results provide new insight to amino acids critical to the transporter function and SNVs that may make their carriers susceptible to altered pharmacokinetics of OATP1B1 substrates.

**Table I:** The SLCO1B1 single nucleotide variants included in the study, their prevalence and predicted consequences of the amino acid changes

SNV	Amino acid change	Variant ID	Frequency^a^	SIFT prediction^b^	PolyPhen prediction^c^
c.227G > A	Gly76Glu	rs866376897	ND	0 (deleterious)	1 (propably damaging)
c.521 T > C	Val174Ala	rs4149056	0.133^d^	0 (deleterious)	0.996 (propably damaging)
c.541C > T	Arg181Cys	rs138965366	0.0000159^e^	0 (deleterious)	0.991 (propably damaging)
c.578 T > G	Leu193Arg	rs72559746	ND	0 (deleterious)	0.863 (possibly damaging)
c.1007C > G	Pro336Arg	rs72559747	0.0000861^f^	1 (tolerated)	0.044 (benign)
c.1034C > T	Thr345Met	rs61760243	0.000385^d^	0.06 (tolerated	0.523 (possibly damaging)
c.1628 T > G	Leu543Trp	rs72661137	0.00000796^f^	0 (deleterious)	0.951 (propably damaging)
c.1724A > T	His575Leu	rs74700754	ND	0.01 (deleterious)	0.142 (benign)
c.1739G > A	Arg580Gln	rs763991908	0.0000333^d^	0 (deleterious)	0.999 (propably damaging)

ND = not determined.

Frequency = Acquired from the gnomAD browser version 2.1.1 (13) https://gnomad.broadinstitute.org/

b SIFT Human Protein online service (11) (http://sift.jcvi.org/www/SIFT_enst_submit.html).

c the Poly-Phen 2 service (10) (http://genetics.bwh.harvard.edu/pph2/) using the human *SLCO1B1* protein sequence (Uniprot entry Q9Y6L6) as the template.

d Global population.

e European (non-Finnish) and South Asian.

f East Asian.

## Materials and Methods

### Materials

Fetal bovine serum (FBS), Hank’s Balanced Salt Solution (HBSS), Dulbecco’s Modified Eagle Medium (DMEM), Coomassie Plus protein assay reagent and HyClone SfX Insect cell medium were from ThermoFisher Scientific (Waltham, MA, USA). Q5® Site-Directed Mutagenesis Kit was from New England Biolabs (Ipswich, MA, USA). Oligomer (Helsinki, Finland) produced the mutagenesis primers. Toronto Research Chemicals (North York, ON, Canada) provided rosuvastatin hemicalcium and rosuvastatin-d6 sodium salt and DCF was from Santa Cruz Biotechnology (Dallas, TX, USA). Dithiothreitol and iodoacetamide were purchased from Sigma-Aldrich (Saint Louis, MO, USA). The peptide standards for MRM analysis were from JPT Peptide Technologies GmbH, Berlin, Germany. ProteaseMax surfactant, LyC endopeptidase and tosylphenylalanylchloromethyl ketone-treated trypsin were from Promega (Madison, WI, USA). All the other chemicals were purchased from Sigma-Aldrich (Saint Louis, MO, USA).

### Preparation of Plasmids Carrying SLCO1B1 Variant Forms

Q5® Site-Directed Mutagenesis Kit and PCR introduced the single nucleotide variants (SNV) to the reference *SLCO1B1* gene (Genebank™ accession number AJ132573.1) in pENTR221 entry vector using the primers described in Supplementary table I. Sequencing service from GATC Biotech (Constance, Germany) verified the presence of the SNVs in the plasmids (data not shown). Baculoviruses carrying the wild type and mutated *SLCO1B1* genes along with previously cloned gene for enhanced yellow fluorescent protein (eYFP, negative control) (14) were produced as described by Tikkanen et al. (15).

### Cell Culture and Protein Expression

HEK293 cells were cultured in DMEM, high-glucose, GlutaMax culture medium supplemented with 10% FBS at 37 °C, 5% CO_2_. Twenty-four hours prior to transduction with baculoviruses, 0.5 *10^6^ cells were seeded in each well of 48-well plates (either poly-D-lysine coated ThermoFisher Scientific™ Nunc™ or Corning™ CellBind™). Sodium butyrate added with the viruses at a final concentration of 5 mM (in-house optimization, data not shown) stimulated the expression of the proteins.

### Cellular uptake assays

The cellular uptake assay was conducted 48 h post-transduction on a heated (37 °C) orbital shaker plate. 500 µl of transport buffer (HBSS with 4.17 mM NaHCO3 and 25 mM HEPES adjusted to pH 7.4 with NaOH) replaced the medium in the wells for a 3-min preincubation. After the removal of the buffer, uptake began with the addition of 125 µl of test solution containing test compound in transport buffer. Aspiration of the test solution stopped the uptake within the linear uptake phase for each substrate: 2 min for rosuvastatin and 15 min for 2,7-dichlorofluorescein (DCF). An immediate three-time wash with 500 µl ice-cold transport buffer followed. The cells were lysed with 125 µl of appropriate lysis buffer (3:1 methanol–water mixture containing rosuvastatin-D6 as an internal standard for rosuvastatin and 0.1 M NaOH in DCF samples). DCF was quantified by fluorescence measurement of the cell lysates (excitation 500 nm, emission 528 nm, bandwith 5 nm) with multimode microplate reader Varioskan LUX (Thermo Fisher Scientific, Vantaa, Finland). The quantification of rosuvastatin is described in the following paragraph. The total protein was determined by mixing 10 µl of cell lysate with 300 µl Coomassie Plus reagent followed by absorbance analysis (595 nm) with Varioskan LUX.

Uptake of test substrates was normalized to total protein amount. Uptake into eYFP expressing cells (representing passive influx) was subtracted from uptake into OATP1B1 expressing cells yielding active OATP1B1 mediated transport. This transport activity in the OATP1B1 variant cells was then normalized to the wild type OATP1B1 cells.

### Rosuvastatin LC–MS/MS Analysis

Liquid chromatography (LC) mass spectrometry (MS) measurements of rosuvastatin were carried out with Waters ultra-high pressure LC–MS/MS instrument (Waters, MA, USA). Rosuvastatin D6 served as an internal standard (ISTD). Liquid chromatography coupled with a Waters UPLC HSS T3 (1.8 μm, 2.1 × 75 mm) column at 40 °C (1.5 µl injection volume) separated the analytes. Mobile phase consisted of 0.1% of formic acid (Merck, Darmstadt, Germany) in ultrapure water (A) and 100% of LC–MS grade acetonitrile (Honeywell, Seelze, Germany) (B). Gradient elution started with 20% of B at 0–0.2 min, continued with 20–95% B at 0.2–1.6 min and the complete run time was 5.5 min including column wash and equilibration with flow-rate as 0.3 mL/min.

The MS measurements were carried out using Waters Xevo TQ-S triple quadrupole mass spectrometer coupled with an electrospray ionization (ESI) on a positive mode. Optimized ms-parameters were: capillary 3.5 kV, cone voltage 84 V for rosuvastatin and 62 V for ISTD, source temperature 150 °C and desolvation temperature 500 °C. Nitrogen was used as a desolvation gas (900 L h − 1) and a cone gas (150 L h − 1), argon as a collision gas. Multiple reaction monitoring quantified the analytes. The precursor and fragment ions for rosuvastatin were 482.19 > 258.12 (collision energy (CE) 32 V) and 482.19 > 300.19 (CE 34 V). For ISTD the ions were 488.17 > 264.17 (CE 30 V) and 488.17 > 306.24 (CE 38 V). The resulting data was analysed with Waters MassLynx V4.1 software.

### Immunofluorescence Staining and Confocal Microscopy

The samples were prepared as described in Sjöstedt et al*.* (16) The used primary antibody (NB100-74,482, Novus Biologicals, Centennial, CO, USA) was used at a dilution of 1:500 in blocking solution and the secondary fluorescent antibody goat anti-mouse IgG (H + L)-Alexa Fluor 488 (ThermoFisher Scientific, USA) was used at a 1:200 dilution in blocking solution. 4′,6-diamidino-2-phenylindole (DAPI) at a concentration of 25 μg/ml visualized the nuclei. The variants were divided into to two batches and due to equipment failure, the microscope type needed to be switched between batches. The used microscopes were Leica TCS SP5 confocal microscope or Aurox Clarity laser free confocal (Aurox Ltd, Oxfordshire, UK) on Leica DM6000 microscope (Leica Microsystems, Wetzlar, Germany). The images were processed with Corel Paintshop Pro (version 23.1.0.27, Corel Corporation, Ottawa, ON, Canada). The immunofluorescence analysis was repeated on a new batch of HEK293 cells transduced with a separate batch of baculoviruses with one batch presented in these figures.

### Crude Membrane Extraction

HEK293 cells were cultured in T175 flasks for 24 h prior to addition of sodium butyrate (5 mM final concentration) and baculoviruses containing either wild type or variant *SLCO1B1*. The cells were collected 48 h later and centrifuged (3000* g*, 15 min). The cells were broken down with Dounce tissue homogenizer, resuspended in Tris-sucrose (TS) buffer (10 mM Tris-HEPES, 250 mM sucrose, pH 7.4) and held on ice. A 30-min centrifugation (3550 g, 4 °C) followed. The supernatant was separated and a further centrifugation step (21,000* g*, 4 °C, 99 min) resulted in a pellet containing the crude membrane. The protein sample was suspended in TS buffer and quantified as described previously.

### LC–MS/MS Based Quantitative Targeted Absolute Proteomics (QTAP) Analysis

The absolute protein expression of OATP1B1 in the crude membrane preparations was quantified using LC–MS/MS-based QTAP approach. The protein sample preparation and quantitation with targeted LC–MS was based on the method described by Uchida et al. (17). Briefly, 50 µg of crude membrane preparations were solubilized, denatured with denaturing buffer, reduced with dithiothreitol and S-carbamoylmethylated with iodoacetamide. Then the alkylated proteins were precipitated with a methanol:chloroform:water mixture. Subsequent precipitates were then dissolved in 6 M urea in 100 mM Tris–HCl (pH 8.5) and diluted fivefold with 100 mM Tris–HCl (pH 8.5). 150 pmol of isotope-labeled peptide mixture (Supplemental table II) (SpikeTides, JPT Peptide Technologies GmbH, Berlin, Germany) serving as internal standard and ProteaseMax surfactant (final concentration 0.05%) were added and the sample was then digested twice: the first with 1/100 LysC endopeptidase and then with 1/100 TPCK-treated trypsin.

The resulting peptides in the samples were analyzed with 6495 QQQ MS (Agilent Technologies, Santa Clara, CA, USA) with 1290 HPLC system (Agilent Technologies) and AdvanceBio peptide Map Column, 2.7 µm, 2.1 × 250 mm (Agilent Technologies) using HPLC method described earlier (18). MS conditions were: 30 µl injection volume, ESI positive ion mode, the source temperature 210 °C, drying gas (nitrogen) flow rate 15 L/min, nebulizer pressure 45 psi, the MS capillary voltage 3 kV and dwell time 40 ms.

The unique peptides for OATP1B1 and Na^+^/K^+^-ATPase were selected according to the in silico peptide selection criteria published by Uchida et al., 2013 (17) and four to six MRM transitions were quantified for each isotope-labeled and unlabeled peptide (Supplementary Table II) (19). The SNVs did not alter the peptides in this sequence. Three transitions with the highest intensity were selected and the peak area ratios of the analyte peptides and their respective internal standards were compared using Skyline application (MacCoss Lab Software, Seattle, WA). The results of OATP1B1 expression are presented as relative to the Na^+^/K^+^-ATPase expression level and normalized to wild type.

### Data Analysis

Cellular uptake assays were conducted in triplicates in two to four separate experiments. The average of these technical triplicates is considered one data point and the data are presented as their mean ± SEM. The proteomics samples (presented as mean ± SEM) were prepared from four and immunofluorescence analysis from two separate batches of HEK293 cells.

The homogeneity of variance in the results was established with Levene’s test (IBM SPSS Statistics for Windows, Version 27.0. IBM Corp, Armonk, NY, USA). Based on these results, the statistical significance of the differences in activity and expression levels was determined with either one-way analysis of variance (ANOVA) with the Dunnett’s post hoc test for multiple comparisons or Kruskal–Wallis one-way analysis of variance with Dunn’s post hoc test for multiple comparisons (GraphPad Prism 6.05, GraphPad Software, San Diego, CA, USA). Maximum velocity (V_max_) and Michaelis–Menten constant (K_m_) values of rosuvastatin transport for selected variants were calculated with non-linear regression from concentration‐dependent assay data and extra-sum-of-squares F-test assessed their statistical significance. P-values below 0.05 were considered significant in all analyses.

## Results

A selection of nine naturally occurring SNVs of OATP1B1 were expressed in HEK293 cells and their effect on transport function was evaluated using two substrates. In addition, LC–MS/MS-based QTAP approach and immunofluorescence analysis was utilized to assess any changes in protein abundance or cellular localization of OATP1B1.

### 2,7-dichlorofluorescein Cellular Uptake Assay

**Fig. 2:**
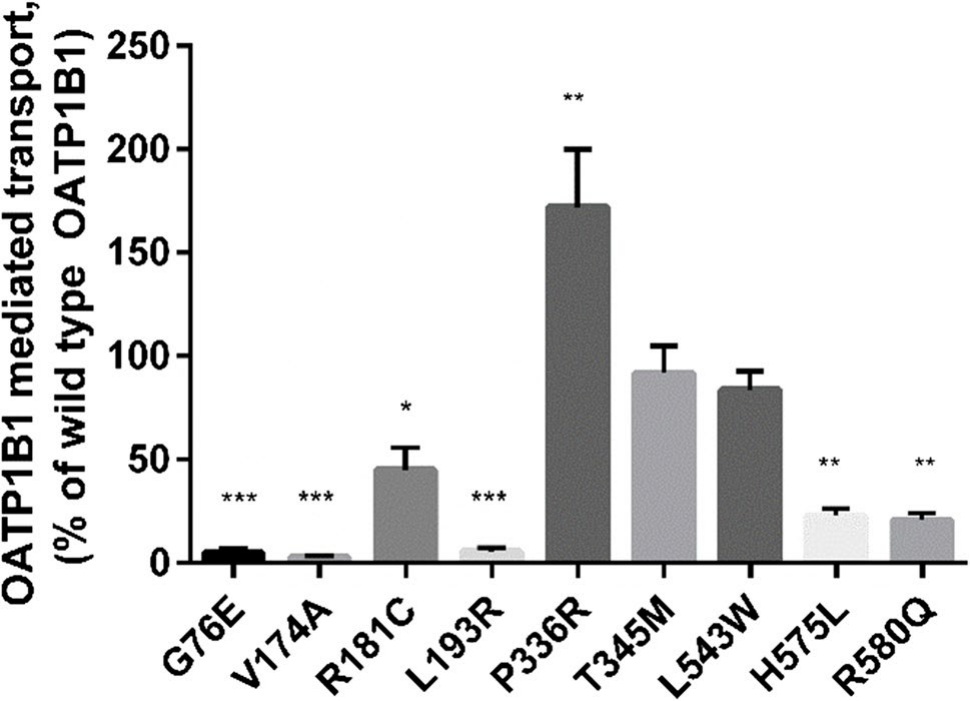
OATP1B1 mediated transport of 1 µM DCF into variant OATP1B1 expressing HEK293 cells over 15 min. Results are calculated as averages of four independent experiments conducted in triplicates and represented as % of wild type OATP1B1 transport ± SEM (n = 4). Statistical difference to the wild type is indicated as: *** = P ≤ 0.001, ** = P ≤ 0.01, * = P ≤ 0.05

The transport activity of the variants was first evaluated with 1 µM DCF cellular uptake assay. DCF transport by G76E, V174A and L193R variants was nearly abolished, with activity less than 10% of wild type OATP1B1 (Fig. 2). Additionally, H575L and R580Q expressing cells showed significantly reduced activity: less than 25% of wild type.

The activity of R181C variant was also reduced significantly, but it was still roughly half of the wild type. T345M and L543W variants showed no significant alteration, while in P336R expressing cells transport was on average 172% of the wild type OATP1B1 transport.

### Rosuvastatin Cellular Uptake Assay

**Fig. 3:**
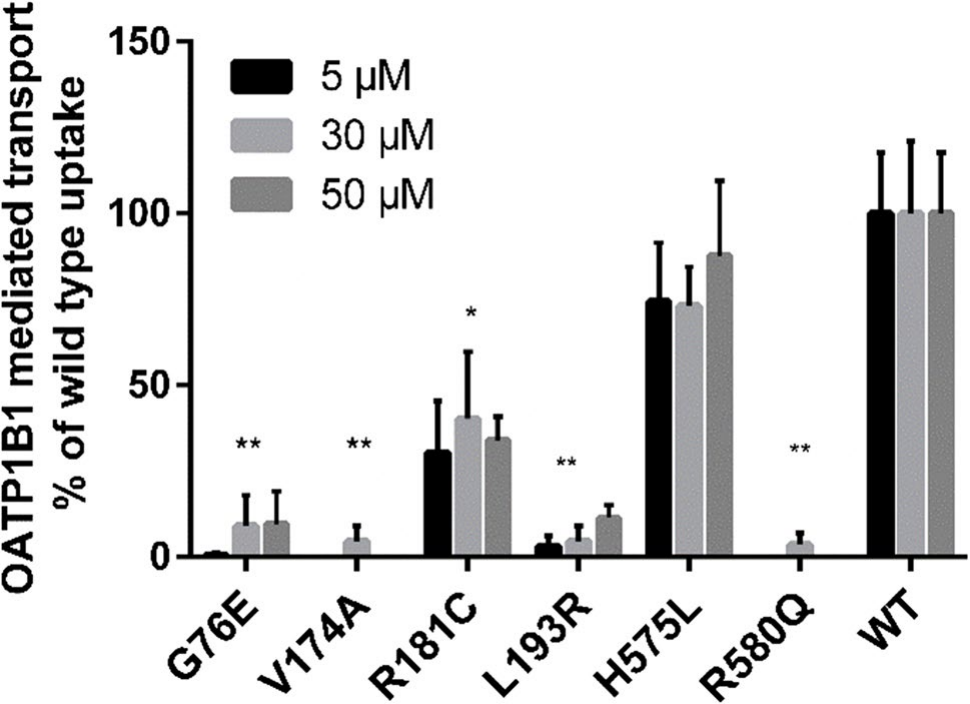
OATP1B1 mediated transport of rosuvastatin into variant OATP1B1 expressing HEK293 cells in 2 min. Results are calculated as averages of two to four experiments conducted in triplicates and represented as % of wild type OATP1B1 transport ± SEM (n = 2–4). Statistical difference to the wild type is indicated as: ** = P ≤ 0.01, * = P ≤ 0.05

**Fig. 4:**
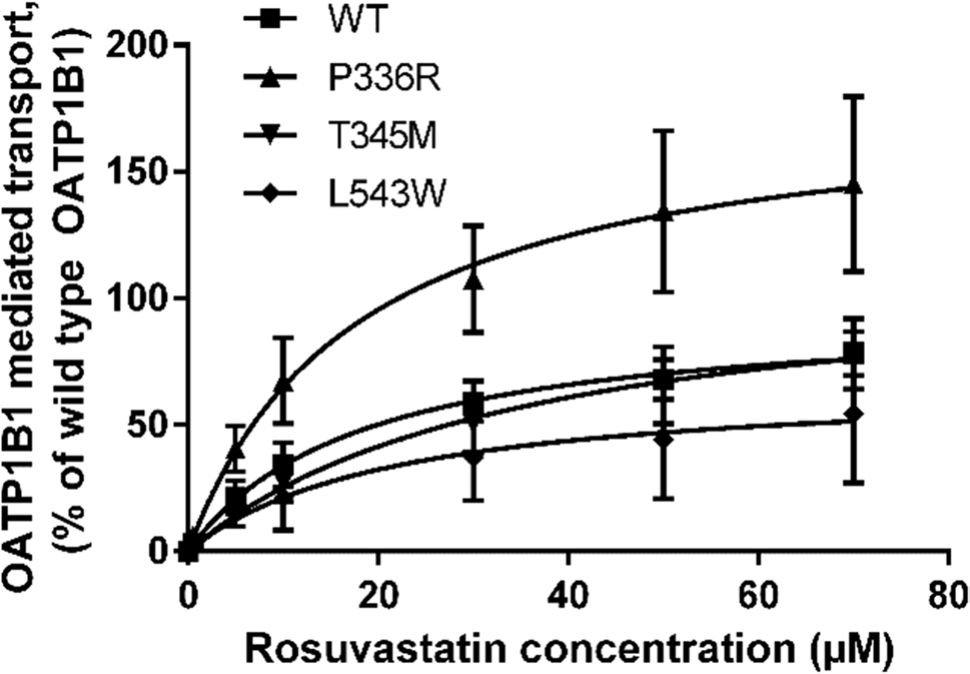
Kinetics of OATP1B1 mediated rosuvastatin transport into variant OATP1B1 expressing HEK293 cells for 2 min. Results are calculated as averages of three experiments conducted in triplicates and represented % of wild type (WT) OATP1B1 transport ± SEM (n = 3)

For evaluation with a clinically relevant OATP1B1 substrate, rosuvastatin, the variants were divided into two groups. The six variants exhibiting reduced cellular uptake of DCF were tested for rosuvastatin transport with three concentrations (5, 30, 50 µM) (Fig. 3) and the rest were subjected to full concentration dependency assay (0.5–70 µM) (Fig. 4).

Much like with DCF transport, G76E, V174A, L193R and R580Q variants caused over 90% reduction in transport of rosuvastatin compared to the wild type (Fig. 3). No active transport was observed in V174A and R580Q expressing cells tested with 5 and 50 µM rosuvastatin. The R181C variant performed similarly in transport of both test substrates, being significantly lower than in the wild type expressing cells. Curiously, the H575L variant transported rosuvastatin better than DCF. Increase in rosuvastatin concentration resulted in no clear change in any of the studied variants.

The P336R variant transported rosuvastatin at a significantly higher velocity with V_max_ 180% of the wild type, P < 0.01 (Fig. 4). T345M substitution led to no significant change in transport velocity and while L543W reduced transport, resulting in a V_max_ value of 67% compared to the wild type, this was not statistically significant. There was no significant change observed in the K_m_ values of the variants compared to the wild type (Table II).

**Table II:** Compilation of results. Uptake and abundance data are normalized to wild type (WT). Maximum velocity of transport (V_max_) of the tested variants is normalized to the calculated V_max_ of WT OATP1B1

Variant	1 µM DCF uptake (%WT) ± SEM	5 µM Rosuvastatin uptake (%WT) ± SEM	Rosuvastatin K_m_^a^ (µM) (95% CI)^b^	Rosuvastatin V_max_^c^ (% WT) (95% CI)		OATP1B1 abundance (%WT) ± SEM
WT	100	100	18.7 (9.9 to 27.6)		100	100
G76E	4.7 ± 2.5	0.5 ± 0.5	ND	ND		24.8 ± 5.5
V174A	2.6 ± 0.8	0 ± 0	ND	ND		9.7 ± 1.7
R181C	45.2 ± 10.5	30.3 ± 15	ND	ND		71.1 ± 24.9
L193R	5.46 ± 1.9	3 ± 3	ND	ND		29.8 ± 4.6
P336R	172 ± 28	216.7 ± 47.8	17.7 (0.0 to 37.9)	179.9 (110.3 to 249.5)		101.4 ± 35.9
T345M	92.1 ± 12.8	71.7 ± 6.8	35.2 (0.0 to 78.8)	114.3 (51.1 to 177.5)		65.1 ± 22.3
L543W	83.9 ± 8.9	51.5 ± 29.9	21.6 (0.0 to 78.0)	67.3 (3.1 to 131.4)		47.2 ± 9.9
H575L	22.8 ± 3.4	74.3 ± 17.2	ND	ND		58.7 ± 20.6
R580Q	20.8 ± 3.3	0 ± 0	ND	ND		36 ± 3.5

ND = not determined.

a = Michaelis–Menten coefficient.

b = 95% confidence interval.

c = maximum uptake velocity.

### Quantitative Targeted Absolute Proteomics Analysis

**Fig. 5:**
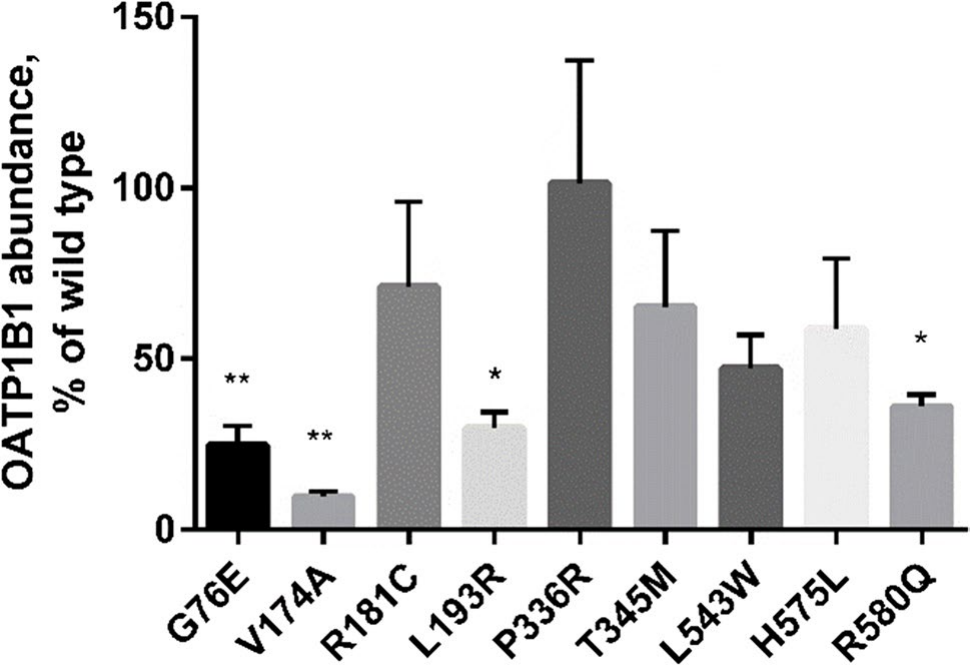
LC–MS/MS proteomics analysis of 50 µg HEK293 crude membrane preparations expressing wild type or variant OATP1B1. Abundance of OATP1B1 was quantified in 4 independent samples, normalized to Na^+^/K^+^-ATPase and compared to wild type (100%) and presented as average ± SEM (n = 4). ** = P ≤ 0.01, * = P ≤ 0.05

Crude membrane protein preparations of the wild type or variant OATP1B1 expressing HEK293 cells were analyzed using LC–MS/MS based quantitative targeted absolute proteomics (QTAP) approach to quantify OATP1B1. After normalization to Na^+^/K^+^-ATPase, the abundance of OATP1B1 in the variant samples were compared to the wild type (Fig. 5). The absolute amounts of OATP1B1 in the samples is listed in Supplementary table III.

**Fig. 6:**
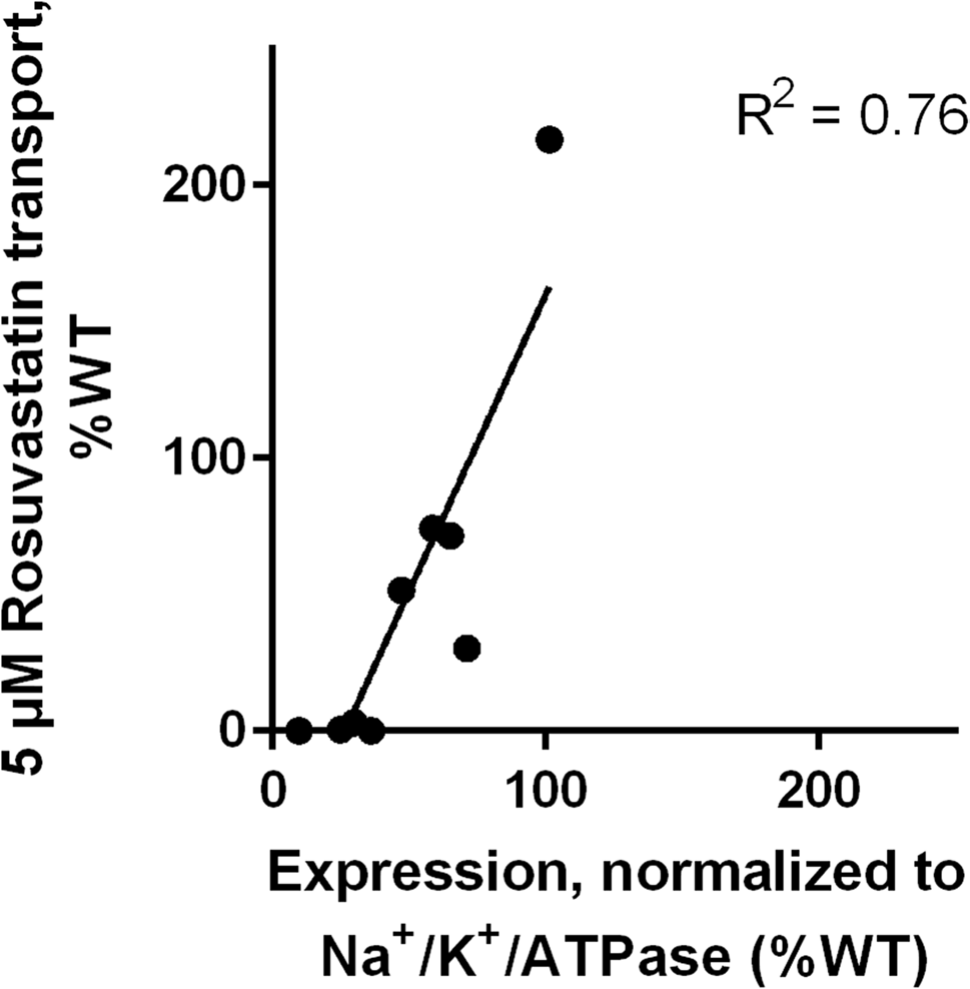
Abundance of OATP1B1 in crude membrane preparations compared to the observed OATP1B1 mediated rosuvastatin transport. The line shows linear regression

With the exception of P336R, all of the studied SNVs resulted in reduced expression of OATP1B1 in HEK293 cells (Fig. 5). G76E, V174A, L193R and R580Q variant samples had the lowest (10–40% of wild type, statistically significant) amount of OATP1B1 protein. The protein levels were compared to the transport activity of the variants and plotted in Fig. 6.

### Immunofluorescence staining and confocal microscopy

**Fig. 7:**
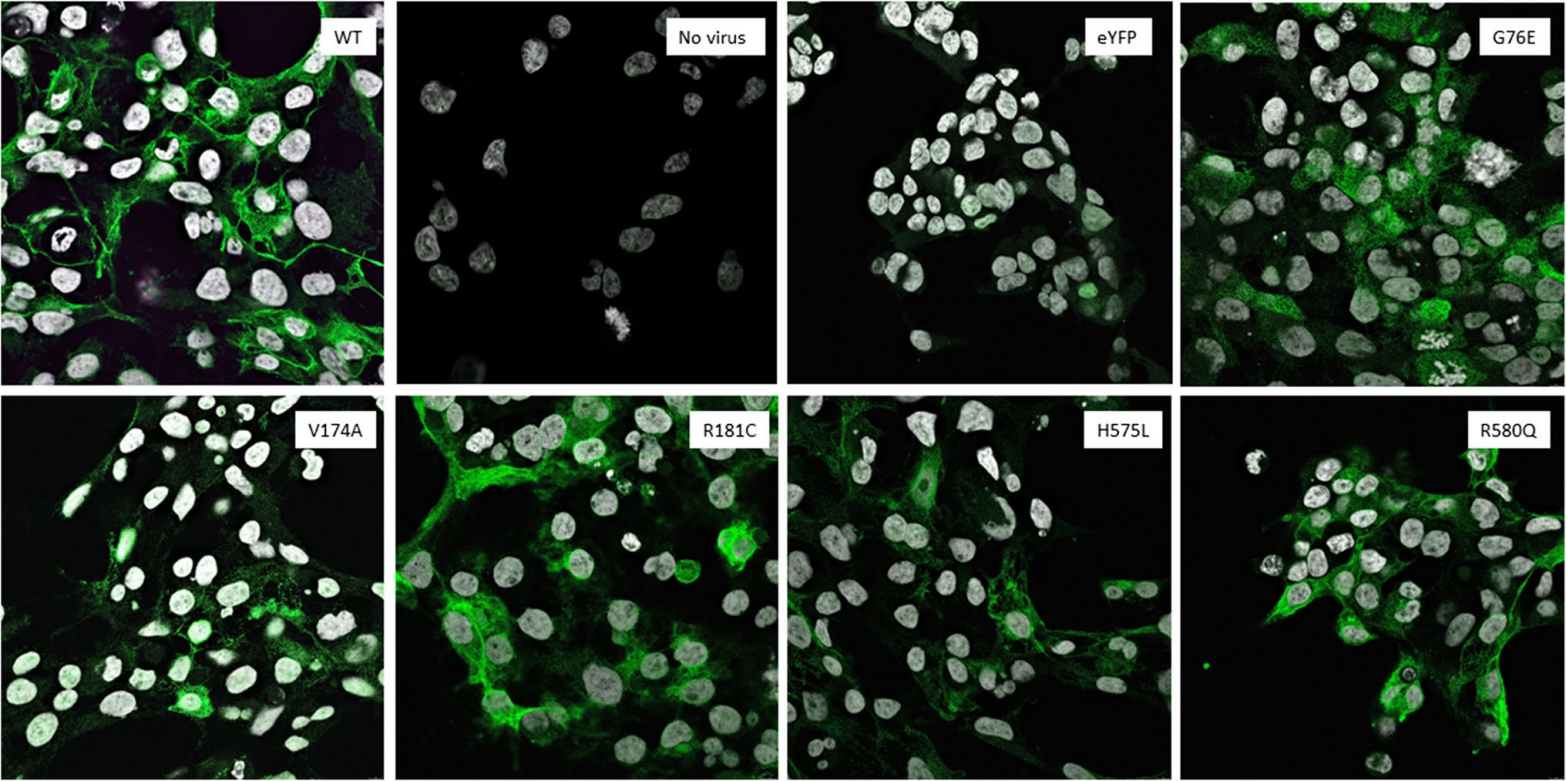
Localization and expression of OATP1B1 protein in representative baculovirus transduced HEK293 cells. OATP1B1 was labeled with mouse NB100-74,482 primary antibody and detected with goat anti-mouse AlexaFluor488 secondary antibody (green) using Leica TCS SP5 confocal microscope. The nuclei are visualized with DAPI (grey). Non-transfected (no virus) and eYFP expressing HEK293 cells were used as negative controls

**Fig. 8:**
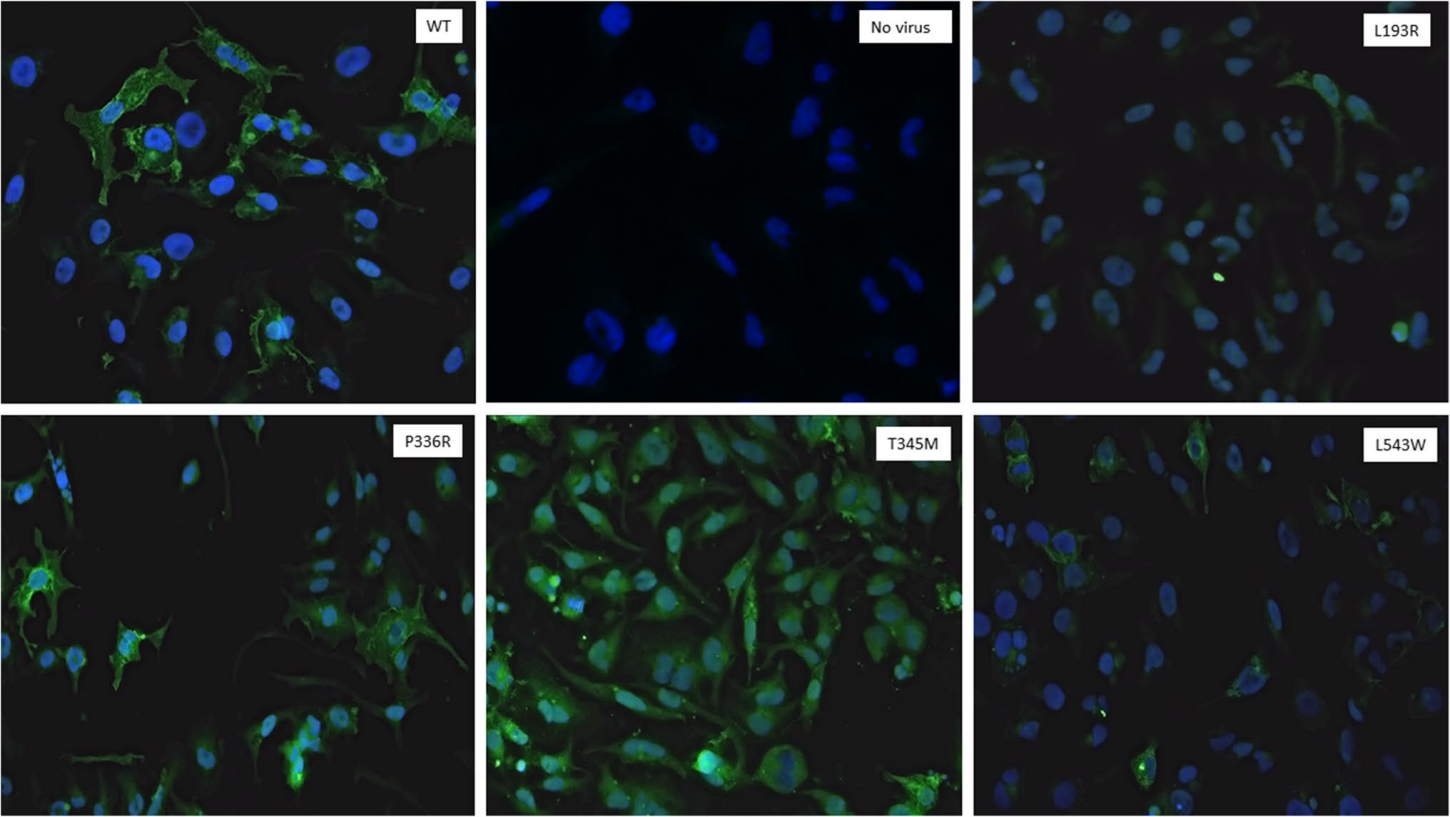
Localization and expression of OATP1B1 protein in representative baculovirus transduced HEK293 cells. OATP1B1 was labeled with mouse NB100-74,482 primary antibody and detected with goat anti-mouse AlexaFluor488 secondary antibody (green) using Aurox Clarity laser free confocal on Leica DM6000 microscope. The nuclei are visualized with DAPI (in blue). Non-transfected HEK293 cells were used as negative control

Immunofluorescence and microscopy provided additional qualitative information on the localization of the OATP1B1 protein in control (non-transduced or eYFP expressing HEK293), wild type (WT) and variant OATP1B1 expressing HEK293 cells. Wild type OATP1B1 virus transduced cells showed typical concentrated band-like green color (Figs. 7 and 8). No green label was present in non-transduced HEK293 cells. The green color in the eYFP expressing cell sample is likely to be derived from the expressed fluorescent protein.

In the G76E expressing cells, OATP1B1 was abundant yet it was not concentrated in similar band-like manner as in the wild type cells. In V174A, H575L, L193R and L543W variant cells the expression of OATP1B1 protein is appears lower than in the wild type (Fig. 7 and 8). Instead, in the R181C, R580Q, P336R and T345M variants the abundance and localization of OATP1B1 appear comparable to the wild type (Fig. 7 and 8).

### Discussion

In this study, OATP1B1 transport activity and localization of nine naturally occurring SNVs were characterized *in vitro*. G76E, R181C, T345M, H575L and R580Q were, to our knowledge, characterized for the first time. A trend (linear regression R^2^ 0.76) was observed between protein expression levels and activity for the studied variants: all loss of function variants had levels below 50% of wild type OATP1B1 (Fig. 6). This does not, however, fully explain the transport activities: for example P336R enhanced transport activity, despite similar abundance of OATP1B1.

In order to compensate for the variability in the used expression system, we used at least two independent batches of baculoviruses in the experiments and performed several independent experiments. Virus titer or multiplicity of infection (MOI) was not determined. Instead, the comparability of the variants was controlled by producing them at the same time with the wild type baculoviruses with standardized amount of DNA in each batch and the variants were only compared to the batch specific wild type virus. The amount of virus was tittered in in-house optimization experiments to achieve maximum transduction and the same ratio was used in all the experiments. The same protocol has previously been used by Sjöstedt et al*.* (16). We performed several experiments with different virus batches and given the low variability level of the results (Fig. 2 and 5), we are confident that the observed changes in the transporter function and expression are due to these SNVs and not variation in the transduction. Phenol red in known to inhibit OATP1B1 (20). While it is included in the cell culture medium, the medium in removed and its traces washed away with phenol red free transport buffer prior to initiation of uptake and consequently should not interfere with the assay.

The transmembrane helix 2, where G76E is located in, was previously shown to be important for transporter localization and function. *SLCO1B1* c.211G > A, a variant that changes glycine to arginine at position 71, reduces OATP1B1 mediated methotrexate transport severely *in vitro* and is associated with reduced methotrexate clearance (7). Substitutions of amino acids to alanine at positions 70, 73, 74 and 76 reduced *in vitro* transport of estrone-3- sulfate (E3S) significantly (21). Similarly, in our study the transport of both DCF and rosuvastatin into G76E variant cells was abolished and immunofluorescence staining showed altered localization compared to the wild type. Moreover, a nearly 75% reduction in OATP1B1 protein abundance was observed. The negative charge of glutamate substitution is more drastic than the previously mentioned alanine substitution, and it is reasonable to assume that both impair folding of the protein into its correct secondary structure. Taken altogether, the data provide evidence that the glycine in position 76 is vital.

Three of the studied variants are located to the putative transmembrane helix 4. The *SLCO1B1* c.521 T > C (V174A) genotype has resulted in increase in plasma AUC levels of e.g. rosuvastatin (22, 23). The *in vitro* data on this variant, nonetheless, is more conflicting. Semi-quantitative Western blot analysis of liver crude membrane fractions of heterozygote c.521 T > C patients showed no difference in OATP1B1 abundance compared to reference (24). On the other hand, liver samples of the same genotype in another study analyzed with LC–MS/MS showed moderate decrease in OATP1B1 abundance compared to reference (8). It should be noted though, that the heterozygote genotype is not comparable to over-expressing systems, which correspond to the homozygote genotype. Nevertheless, the data from over-expressing systems is also contradictory. The cell-surface expression level of V174A was decreased in Western blot of biotinylated HeLa cell samples (25) and in immunocytochemical analysis of HEK293 and HeLa cells (26), while no change was observed in Western blot analysis of HEK293 and HeLa (both whole cell and biotinylated) samples in other studies (27–29). Differences in these results can arise from used cellular expression system, sample preparation and analysis methods. To our knowledge, this study is the first to quantify OATP1B1 V174A in an over-expressing system with a LC–MS/MS proteomics method. We observed > 90% decrease in protein amount in the crude membrane fractions of OATP1B1 V174A compared to the wild type, which is in accordance to a meta-analysis, where the relative abundance of V174A OATP1B1 was 37% of the wild type OATP1B1 (30).

The *in vitro* transport activity of V174A has also varied between studies. The transport of E3S and estradiol-17β- glucuronide (E217G) was not altered in two studies (27, 28) while it was reduced in others, along with rosuvastatin transport (25, 31). For the most part, the results have been consistent with the expression levels in those studies. Crowe et al*.*, however, reported significantly lower accumulation of E217G in V174A expressing HEK293 cells but no change in cell surface expression (29). In our study, the activity was abolished with both tested substrates and interrelated with the protein expression levels.

The arginine in position 181, also located in transmembrane helix 4, is conserved throughout the OATP1 family (32). In a previous study, lysine substitution at this position resulted in 90% reduction of OATP1B1 expression, while the reduction with alanine and histidine substitution was only 20 and 40%, respectively (33). In our study, the protein expression of R181C was reduced by 30% compared to the wild type and transport of both tested substrates was less than 50% of wild type. Localization in immunofluorescence samples, instead, appeared comparable to wild type. These results suggest that the arginine is more important for the transport function of OATP1B1 than for stable protein expression.

L193R, the third of the studied variants in transmembrane helix 4, reduced the transport of both tested substrates severely, and the amount of variant protein was only 40% of the wild type. Immunofluorescence results also suggest lower abundance and plasma membrane localization compared to the wild type. This is in line with previous characterization of L193R, where the transport of sulfobromophthalein, E217G and cholyltaurine was abolished and Western blot analysis showed markedly reduced localization in crude membrane samples of both transfected MDCKII cells and a human liver sample (34).

The SNVs exhibiting increased or unaltered activity (P336R and T345M) are both found in the putative transmembrane helix 7 (Fig. 1). T345M did not alter either the activity or the localization or abundance of OATP1B1. Proline at the position 336 is a part of the NPxY motif involved in sorting of basolateral membrane proteins (35). This motif is conserved in OATP1B1, OATP1B3, OATP1C1 and OATP1A2. Alanine substitution decreased transport activity moderately (35). Clinical data on P336R is sparse: a single subject with the P336R variant had a high pravastatin AUC value and low total and non-renal clearance compared to the reference genotype (36). *In vitro* P336R substitution did not change or only slightly enhanced the transport of E3S, pravastatin, atorvastatin, cerivastatin and simvastatin (26). In our study, however, the activity of the P336R variant was significantly increased: DCF transport was 172% and the V_max_ of rosuvastatin transport was 180% of wild type. The protein amount, however, did not differ from the wild type and the localization of the variant appeared comparable to wild type. The increase in V_max_ without an increase in K_m_ or expression levels suggests that this variant has a higher catalytic turnover rate than the wild type OATP1B1.

L543W is the only variant in our study that is located to transmembrane helix 10 (Fig. 1). It was first discovered in a Japanese man suffering from rhabdomyolysis (37). In our study, the uptake of DCF was not altered and rosuvastatin V_max_ was reduced moderately. This in accordance with the first *in vitro* characterization, where this variant did not alter the uptake of E3S but reduced pravastatin V_max_ (no change in K_m_) (38). Cell-surface biotinylation and Western blot analysis showed normal expression and localization on the plasma membrane in HEK293 cells. Our QTAP analysis, conversely, revealed reduced (47%) protein expression compared to the wild type. This was also apparent in the immunofluorescence samples.

H575L and R580Q, are found in transmembrane helix 11. Surprisingly, the H575L variant reduced transport activity in a substrate specific manner. The reduction in rosuvastatin transport was in line with reduction in protein abundance, yet DCF transport was more drastically reduced, indicating the possibility of additional changes in substrate recognition or translocation. R580Q impaired the transport of both tested substrates significantly and is likely caused by decreased protein expression. Immunofluorescence, however, suggested no drastic change in neither abundance nor cellular localization of OATP1B1. Previously, alanine substitution at position 580 resulted in over 50% reduction in E3S transport (39). In a Western blot analysis of biotinylated HEK293 plasma membrane samples alanine reduced cell surface expression while lysine or histidine substitution did not change it (33). These results together with our novel findings suggest that a positively charged amino acid is necessary in the 580 position for proper function and localization of OATP1B1.

Overall, the studied variants in transmembrane helices 2, 4 and 11 appear to be crucial for proper membrane localization and function of OATP1B1, while the studied variants in helices 7 and 10 retained much of the function of the wild type OATP1B1.

The clinical significance of the *SLCO1B1* c.521 T > C (V174A) genotype has prompted issuance of clinical prescribing guidelines (40). Individuals with two decreased-function alleles (low function phenotype) are considered to be at high myopathy risk during simvastatin treatment and the treatment is recommended to be initiated with a lower dosage. Based on our transport activity results, SNVs G76E, L193R, R580Q, could be categorized as low function variants, while R181C and H575L could be classified as intermediate function variants. T345M and L543W variants can be considered normal function variants, but P336R might lead to decreased plasma AUC values of rosuvastatin. Individuals with one decreased function allele or two intermediate function alleles could correspond to the intermediate function phenotype (40), which would also warrant caution when prescribing simvastatin. Due to the rarity of these variants (Table I), they are most likely to be found as heterozygotes. They could also be found as haplotypes with other SNVs such as V174A or N130D latter being very common in Sub-Saharan, East Asian and Oceanian populations (6). This could further alter their effects on transport activity. *In vitro* co-expression of P336R with N130D did not alter transport function compared to P336R expression only (26). When expressed with V174A, transport function was slightly improved compared to just V174A. Further research is required to establish the clinical effects of these phenotypes.

## Conclusions

We have determined the transport activity and protein levels of nine variants of OATP1B1. To our knowledge, the G76E, R181C, T345M, H575L and R580Q variants were characterized for the first time. The *in vitro* transport activity of six of the studied variants (G76E, V174A, R181C L193R, H575L and R580Q) was significantly reduced, two (T345M and L543W) had similar activity and one (P336R) variant had enhanced transport activity compared to the wild type OATP1B1. Overall, our results support previous studies suggesting that transmembrane helices 2, 4 and 11 are important for proper membrane localization and function of OATP1B1. While there was a trend between protein amount and activity, protein amount does not fully explain the observed transport activities, indicating involvement in substrate recognition or translocation for some of these residues. G76E, L193R and R580Q caused loss of function and thus could predispose the individuals with these genotypes to adverse effects of OATP1B1 substrate drugs similarly to V174A.

## Funding Statement

We are grateful for the funding provided by Svenska Kulturfonden, Medicinska Understödsföreningen for Liv och Hälsa, DRA Consulting oy, Instrumentarium Science Foundation and Finnish Cultural Foundation. The UEF Metabolomics laboratory is supported by Biocenter Finland and Biocenter Kuopio and the Drug Discovery and Chemical Biology Network and the Light Microscopy Unit are funded by Biocenter Finland. Dr. Melina M. Malinen received funding from the European Union’s Horizon 2020 Research and Innovation program under the Marie Skłodowska-Curie grant agreement number 799510.

## Supplementary Information

Below is the link to the electronic supplementary material.

Supplementary file1 (DOCX 20.3 KB)
