# Supplementary material for: The Effect of Single Nucleotide Variations in the Transmembrane Domain of OATP1B1 on in vitro Functionality
Source: Pharm Res. 2021 Oct 13;38(10):1663–75. doi: 10.1007/s11095-021-03107-8 (PMC8602229; doi:10.1007/s11095-021-03107-8)
Supplement: Supplementary file 1 — Supplementary file1 (DOCX 20.3 KB) [file 11095_2021_3107_MOESM1_ESM.docx]

“The effect of single nucleotide variations in the transmembrane domain of OATP1B1 on *in vitro* functionality.”

Kiander Wilma, Vellonen Kati-Sisko, Malinen Melina M, Gynther Mikko, Hagström Marja, Bhattacharya, Madhushree, Auriola Seppo, Koenderink Jan B, Kidron Heidi.

**Supplementary material**

Contents:

**Supplementary table I** Mutagenesis primers used in creating SNVs in the SLCO1B1 gene.

**Supplementary table II.** Peptide sequences and MRM transitions (m/z) used for LC-MS/MS analysis of OATP1B1 and Na^+^/K^+^-ATPase.

**Supplementary table III** Absolute amount of OATP1B1 in proteomics samples.

**Supplementary table I** Mutagenesis primers used in creating SNVs in the SLCO1B1 gene. Small case letter denotes the nucleotide substitution.

| SNV | Amino acid change | Forward | Reverse |
| --- | --- | --- | --- |
| c.227G>A | Gly76Glu | TTTGAAATTGaAAATTTGCTTGTGATTG | GCTTCCGTCAATAAAACC |
| c.521T>C | Val174Ala | TGGATATATGcGTTCATGGGTAATATG | CATGTATGACCCAGATTC |
| c.541C>T | Arg181Cys | TAATATGCTTtGTGGAATAGGG | CCCATGAACACATATATCC |
| c.578T>G | Leu193Arg | CCATTGGGGCgTTCTTACATTG | TACTATGGGAGTCTCCCC |
| c.1007C>G | Pro336Arg | CTTACTAATCgCCTGTATGTTATG | GATGCTTTTAAAAGACTGG |
| c.1034C>T | Thr345Met | GTGCTTTTGAtGTTGTTACAAG | AAACATAACATACAGGGG |
| c.1628T>G | Leu543Trp | ATACAAGTCTgGAATTTATTTTTCTCTG | TGCAACAAAAAAGTAAAATTTC |
| c.1724A>T | His575Leu | CTGGGTTTCCtCTCAATGGTTATAC | TGCAAGTGATTTCAATTCAG |
| c.1739G>A | Arg580Gln | ATGGTTATACaAGCACTAGGAGG | TGAGTGGAAACCCAGTGC |

**Supplementary table II.** *Peptide sequences and MRM transitions (m/z) used for LC-MS/MS analysis of OATP1B1 and Na^+^/K^+^-ATPase.*

| **Protein** | **Unlabeled/ Isotope-labeled** | **Peptide sequence** | **Transition number** | **Precursor**  **Ion** | **Product**  **Ion** |
| --- | --- | --- | --- | --- | --- |
| OATP1B1 | Unlabeled | LNTVGIAK | 1 | 408.3 | 702.4 |
|  |  |  | 2 | 408.3 | 588.3 |
|  |  |  | 3 | 408.3 | 487.3 |
|  |  |  | 4 | 408.3 | 218.1 |
|  | Isotope-labeled | LNTVGIAK* | 1 | 412.3 | 710.4 |
|  |  |  | 2 | 412.3 | 596.4 |
|  |  |  | 3 | 412.3 | 495.3 |
|  |  |  | 4 | 412.3 | 226.1 |
| Na^+^/K^+^-ATPase | Unlabeled | AAVPDAVGK | 1 | 414.2 | 685.4 |
|  |  |  | 2 | 414.2 | 624.3 |
|  |  |  | 3 | 414.2 | 586.3 |
|  |  |  | 4 | 414.2 | 525.3 |
|  |  |  | 5 | 414.2 | 489.3 |
|  |  |  | 6 | 414.2 | 374.2 |
|  | Isotope-labeled | AAVPDAVGK* | 1 | 418.2 | 693.4 |
|  |  |  | 2 | 418.2 | 624.3 |
|  |  |  | 3 | 418.2 | 594.3 |
|  |  |  | 4 | 418.2 | 525.3 |
|  |  |  | 5 | 418.2 | 497.3 |
|  |  |  | 6 | 418.2 | 382.3 |

*denotes labelled lysine (K) with a stable isotope ^13^C and ^15^N

**Supplementary table III** *Absolute amount of OATP1B1 in proteomics samples.*

| Variant | Average OATP1B1 abundance fmol/µg protein | Standard error of the mean |
| --- | --- | --- |
| WT | 1.235 | 0.307 |
| G76E | 0.425 | 0.271 |
| V174A | 0.155 | 0.051 |
| R181C | 0.855 | 0.330 |
| L193R | 0.335 | 0.127 |
| P336R | 2.025 | 1.181 |
| T345M | 0.583 | 0.167 |
| L543W | 0.593 | 0.185 |
| H575L | 0.598 | 0.164 |
| R580Q | 0.745 | 0.373 |
